# Identification and analysis of odorant receptors expressed in the two main olfactory organs, antennae and palps, of *Schistocerca americana*

**DOI:** 10.1038/s41598-022-27199-3

**Published:** 2022-12-31

**Authors:** Alejandra Boronat-Garcia, James Iben, Eunice Dominguez-Martin, Mark Stopfer

**Affiliations:** 1 Section on Sensory Coding and Neural Ensembles, National Institutes of Health, Eunice Kennedy Shriver National Institute of Child and Human Development, Bethesda, MD USA; 2 Molecular and Genomics Core, National Institutes of Health, Eunice Kennedy Shriver National Institute of Child and Human Development, Bethesda, MD USA; 3 Biochemistry Section, National Institutes of Health, National Institute of Neurological Disorders and Stroke, Bethesda, MD USA

**Keywords:** Gene expression, Neuroscience, Olfactory system, Olfactory receptors

## Abstract

Locusts depend upon their sense of smell and provide useful models for understanding olfaction. Extending this understanding requires knowledge of the molecular and structural organization of the olfactory system. Odor sensing begins with olfactory receptor neurons (ORNs), which express odorant receptors (ORs). In insects, ORNs are housed, in varying numbers, in olfactory sensilla. Because the organization of ORs within sensilla affects their function, it is essential to identify the ORs they contain. Here, using RNA sequencing, we identified 179 putative ORs in the transcriptomes of the two main olfactory organs, antenna and palp, of the locust *Schistocerca americana*. Quantitative expression analysis showed most putative ORs (140) are expressed in antennae while only 31 are in the palps. Further, our analysis identified one OR detected only in the palps and seven ORs that are expressed differentially by sex. An in situ analysis of OR expression suggested ORs are organized in non-random combinations within antennal sensilla. A phylogenetic comparison of OR predicted protein sequences revealed homologous relationships among two other *Acrididae* species. Our results provide a foundation for understanding the organization of the first stage of the olfactory system in *S. americana*, a well-studied model for olfactory processing.

## Introduction

Olfaction allows animals to detect, identify, and discriminate among hundreds of thousands of odor molecules present in the environment. This ability is essential for animals’ survival and yet requires a complex process to generate the high dimensional neural representations needed to characterize odorant molecules, which have different sizes, shapes and electrical charges, and are often organized into chaotic and turbulent odor plumes^1^. Understanding the anatomical organization of the olfactory system at the cellular and molecular levels has provided important insights into the coding mechanisms underlying olfaction^2–6^, and studies performed in insects have contributed substantially to our knowledge of odor processing^3,7–10^. Further, mechanisms that allow the olfactory system to generate representations for odors have been shown to be widely conserved among very divergent species^11,12^.

Odor molecules are detected by transmembrane proteins including odorant receptors (ORs) expressed in the dendrites of olfactory receptor neurons (ORNs). Insect ORs are members of the seven transmembrane domain (TMD) superfamily and are highly divergent^13–16^. Normally, a single ORN expresses the co-receptor Orco together with only one type of OR which imbues the neuron with its tuning properties and its complex and diverse response dynamics^15,17–19^. In insects, ORNs are housed in specialized hair-like structures called olfactory sensilla which are classified into three morphological types: basiconic, trichoid, and coeloconic^20,21^. Although the principles underlying the organization of ORNs within sensilla are poorly understood, there is evidence to suggest this organization plays important roles in olfactory coding. Sensilla, for example, can be organized into different functional classes based on the combination of ORN types housed within them^5^, and olfactory ephaptic interactions appear to require the close apposition of functionally related ORNs^22^. Understanding ORN organization at the sensilla level will help clarify the earliest stages of odor coding.

The locust *Schistocerca americana* is a well-studied model of olfactory processing, and electrophysiological studies in this species have improved fundamental understanding of information processing by neurons^11,18,23,24^. However, little is known of the organization of ORNs in this organism in part because the ORs expressed in its odorant sensing organs have not yet been identified. Identifying these ORs will, among other benefits, facilitate research into olfactory systems and neural codes for sensory stimuli.

The locust has two main odor sensing organs: the antennae and the mouthparts (maxillary and labial palps). While each antenna houses ~ 100,000 ORNs, each of the four palps contains only ~ 150^25–29^.These two organs have been suggested to play different roles in odor processing, with the antennae likely serving as general purpose odor sensors, and the palps processing food odors^27,30,31^. However, a better understanding of these structures and their functions will require identifying the ORs they express.

To extend our knowledge of OR genes expressed in *S. americana* (Same) we analyzed the transcriptomes of the antennae and the palps. Because female and male locusts are sexually dimorphic and show some different behaviors^32,33^, we separately analyzed tissue from adult females and males. All together, we identified 179 transcripts of putative ORs (named here as SameORs) expressed in the two main olfactory organs. Of the 159 sequences encoding proteins predicted to have at least two transmembrane domains (TMDs), 140 were present at medium to high abundances in the antenna, but only 31 were found in the palps. We found one putative OR (SameOR63) expressed only in the palp, suggesting this receptor plays a palp-specific role. Also, we found some putative SameORs expressed differentially in female and male locusts. In the antenna: four putative ORs (SameOR1, SameOR7, SameOR25, and SameOR152) were significantly more highly expressed in male than female and one (SameOR40) more expressed in females. In the palp: two putative ORs (SameOR33 and SameOR29) were significantly more highly expressed in male than female tissue, suggesting sexual dimorphisms of OR expression. Also, an in situ analysis of OR expression suggested ORs are organized in non-random combinations within antennal trichoid sensilla. Finally, we compared the sequences we obtained for *S. americana* to those of two other related *Acrididae* species. Our results provide a necessary foundation for understanding the organization of the peripheral olfactory system of the locust.

## Results

### Transcriptome sequencing and assembly

From female and male antennae, a total of 101.8 and 107.3 million raw reads, respectively, were produced using Illumina sequencing platforms, and from female and male palps, a total of 646 and 622.8 million raw read pairs, respectively, were produced (Supplementary Table 1 shows the number of read pairs produced for each tissue type sample). GC content ranged between 44 and 46%. After assembling the transcriptome, we obtained a total of 850,000 sequences, with an N50 of 1383 bp, a mean length of 726 bp, and a mapping percentage of reads to the assembly of 83% (Supplementary Table 2 shows additional assembly statistics.) To assess the quality of the assembly (i.e., accuracy and completeness), we performed two standard tests. First, we calculated the TransRate assembly score, which is appropriate for transcriptomes assembled de novo^34^. Our TransRate score was 0.85 indicating high accuracy and completeness according to established standards^34^. Second, we calculated BUSCO, which checks the transcriptome for the presence of a group of single-copy orthologous genes thought to be essential for life and occurring in more than 90% of species^35^. BUSCO results (Supplementary Fig. 1) showed that of the 255 genes evaluated, 249 (97.6%) were complete—from which 89 (34.9%) were complete and single-copy and 160 (62.7%) were complete but duplicate—4 (1.5%) were fragmented, and 2 (0.9%) were missing. Overall, these metrics indicate a high-quality assembly in terms of completeness^36^. The raw reads are deposited in the NCBI SRA database (BioProject ID PRJNA889432).

### Identification of sequences encoding putative odorant receptors

**Figure 1:**
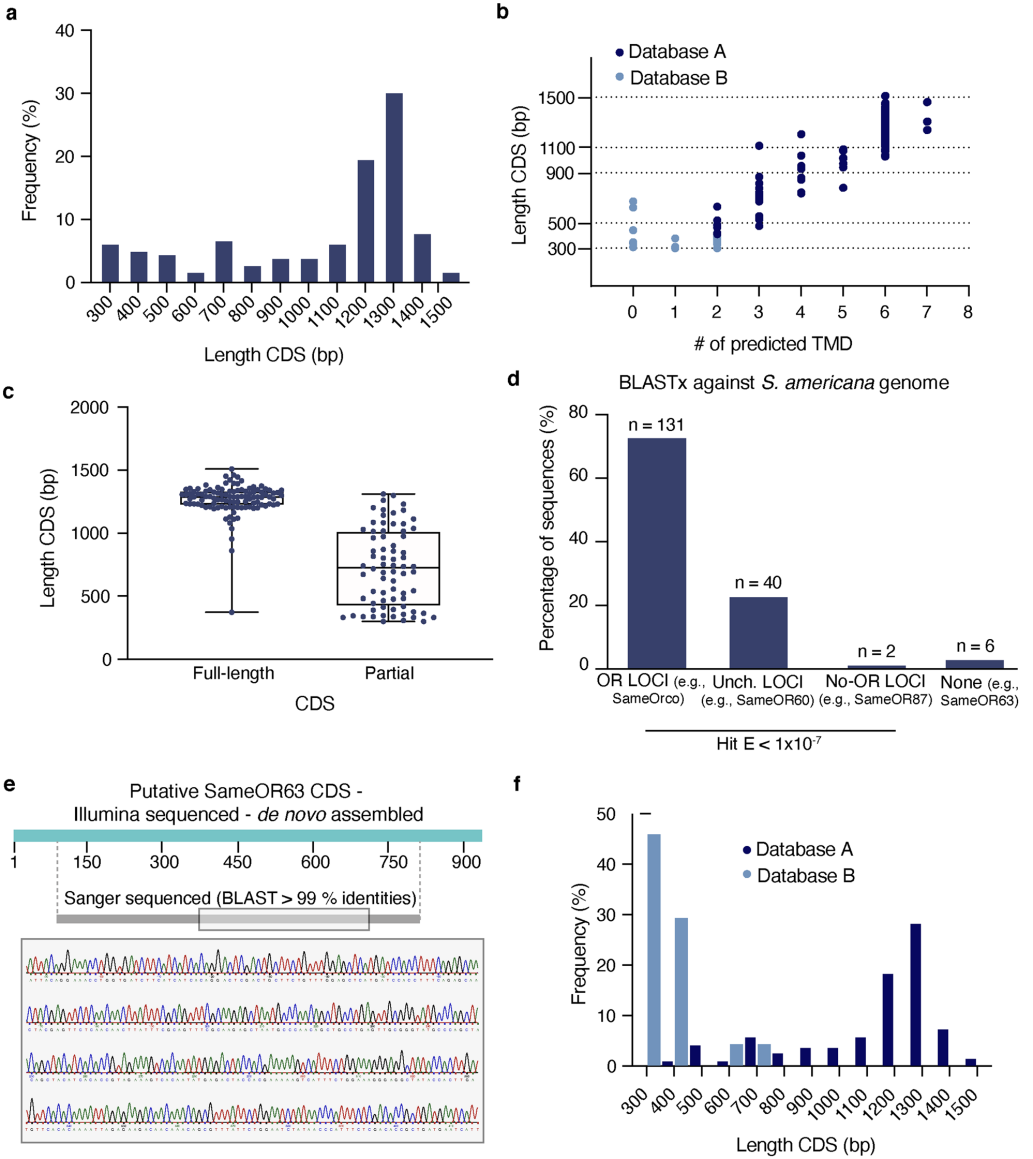
Statistical characterization of SameORs identified in the unified transcriptome assembly. (**a**) Frequency distribution of CDS length (bp). (**b**) Relation between CDS length and number of TMDs predicted from the deduced amino acid sequences of the 179 putative ORs. Putative OR sequences were divided into database A (dark blue) and database B (light blue) for further analysis. (**c**) Length of full and partial CDSs. (**d**) Blastx results of putative SameOR CDSs against a recently posted *S. americana* genome assembly. Shown are the percentage of sequences with a significant match (E < 1E^−7^) to either a predicted OR, an uncharacterized (Unch.) or a none-OR loci, and those that did not match any genomic region (None). (**e**) Schematic highlighting of SameOR63 CDS region amplified by RT-PCR and sequenced using Sanger. The Sanger chromatogram at the bottom is an example of the sequencing results. (**f**) Frequency distributions of CDS length in Database A (dark blue) and Database B (light blue).

We identified a total of 179 transcripts encoding putative ORs in the antennae and/or palps (See Methods and Supplementary Data 1–2). The lengths of the predicted coding sequences (CDS) ranged from 300 to 1509 bp (Fig. 1a). The majority of the CDS (127, or 70.9% of the total) was longer than 900 bp, while 28 (15.6%) were 501–900 bp and 24 (13.4%) were 300–500 bp. Most of the 179 putative ORs (115, 64.2%) had predicted secondary structures with a topology containing six or seven TMDs, 19 (10.6%) had four or five predicted TMDs, 35 (19.5%) had two or three TMDs, and 10 (5.6%) contained one or no TMDs (Fig. 1b, Supplementary Data 1–2). A total of 107 (59.7%) of the 179 sequences had full-length CDS (Fig. 1c) with mean length 1267 bp. Of these 107 full-length sequences, almost all (101) had six or seven predicted TMDs.

We performed BLASTx searches to compare transcripts to an *S. americana* genome recently deposited in NCBI (see Methods). We found most of our sequences (173 of 179) produced a hit to those sequences annotated to be open reading frames (ORFs) in the assembled genome, with an identity ranging from 69.7 to 100% (Supplementary material Excel file S1). Most of our sequences (n = 131) matched against loci predicted to code for ORs in the genome (e.g., SameOrco, SameOR1, SameOR2, SameOR7, SameOR40, SameOR25) while 40 matched uncharacterized loci (e.g., SameOR3, SameOR60, SameOR71). In addition, six of our sequences did not match any locus (e.g., SameOR63, SameOR94, SameOR155, SameOR133, SameOR165, SameOR188) and two matched against non-OR loci (i.e., SameOR80 and SameOR87) (Fig. 1d and Supplementary Data 1–2).

To evaluate the success of our sequence assembly with an independent approach, we designed primers for some of our putative ORs and performed RT-PCR followed by sequencing to check for expression of the identified putative SameOR transcripts in antenna and palp tissues. Among the analyzed sequences, we included some with no matches to the NCBI *S. americana* genome (e.g., SameOR63), some that matched to uncharacterized loci in the genome but with short alignments (e.g., SameOR71 with only 81 bp aligned), and some that matched to a predicted OR loci (e.g., SameOrco). For example, for SameOR63, we amplified ~ 800 bp of the sequence and aligned it to our de novo assembled sequences (Fig. 1e). Our results confirmed the successful assembly of all the tested sequences and showed that all of them code for genes that are expressed in antennae and/or palp tissues (Fig. 1e, Supplementary Fig. 2).

Because ORs are predicted to have seven TMDs^13–16^, we divided our collection of identified transcript sequences in two databases based upon the structural topology predicted from the derived amino acid sequences (Fig. 1b) and CDS quality (see Methods). The higher quality Database A contained 159 putative ORs with a predicted topology ranging from two to seven TMDs, including 115 sequences with six or seven TMDs. Most of Databases A's transcripts (127, 79.9%) were longer than 900 bp, and of these, 105 were full-length sequences, while 26 (16.3%) were 501–900 bp, and only six (3.8%) were 400–500 bp long (Fig. 1f). Database B contained the remaining 20 putative ORs. Most of these were 300–500 bp (18, 90%) and only two (10%) were 501–700 bp (Fig. 1f). The number of predicted TMDs in these sequences ranged from zero to two (Fig. 1b). Because ORs are predicted to have seven TMDs, only sequences in Database A were used for further analyses including the quantification of transcript abundances, differential expression and spatial distribution analyses, and the evolutionary relationships among related species.

### Tissue specific expression and sex differences of putative ORs in the antennae and palps

**Figure 2:**
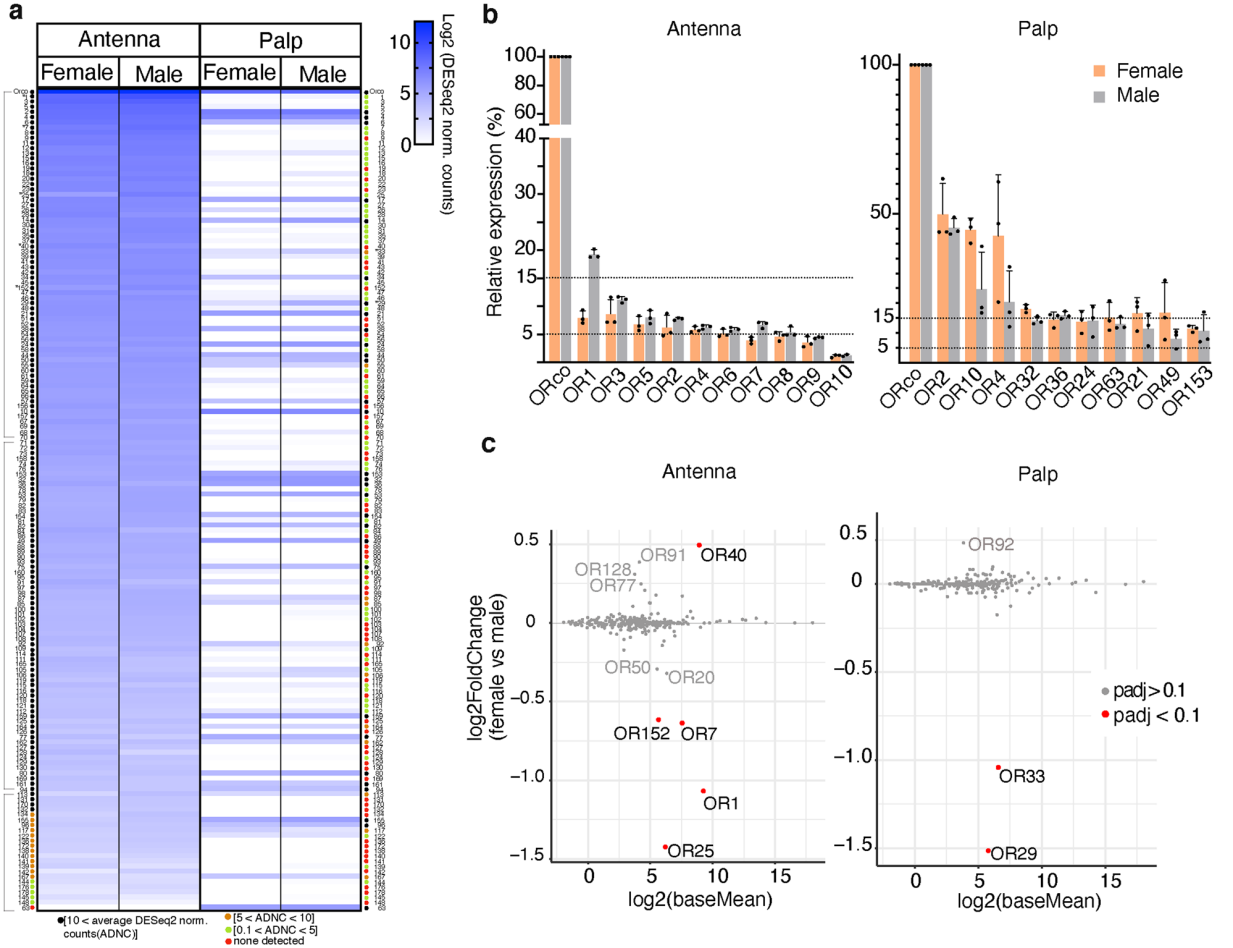
Expression of putative SameORs by tissue type and sex. RNAseq reads were aligned against identified putative SameORs and quantified. All data shown are based on DESeq2 normalized read counts. Quantification was performed in three replicates per condition. (**a**) Expression heat map of the 159 putative SameORs contained in Database A. Data are sorted according to antenna expression from highest to lowest normalized counts. Each row corresponds to the same putative SameOR in all columns. Colored circles next to each named SameOR indicate expression level: black, 10 < average <  ~ 4300; orange, 5 < average < 10; green, 0 < average < 5 normalized counts. Red: none detected. Light gray lines indicate grouped OR labels; see Supplementary Fig. 3 for an enlarged version. Asterisks: statistically significant differences in expression between female and male in antenna or palp tissues from panel (**c**). (**b**) Expression of the 10 most expressed putative SameORs in female (orange bars) and male (gray bars) tissue. Expression level is given relative to Orco. Error bars: standard deviation. (**c**) Differential expression between female and male in antennae (left panel) and palp (right panel) tissues. Red: differentially expressed putative SameORs padj < 0.1.

To determine whether the identified putative ORs were expressed differentially in antenna and palp, we quantified in both tissues the number of reads of the 159 putative ORs in Database A. Because the palp contains many fewer ORNs than the antenna, to increase sensitivity we performed additional reads from the palp (antenna: ~ 100 M reads; palp: ~ 650 M reads; see Methods). In the antenna tissue samples, 140 out of the 159 putative ORs were present in medium to high abundance (above 10 DESeq2 normalized counts on average per tissue, Fig. 2a black color labels left panel, Supplementary Figs. 3–4), one putative OR was not detected (Fig. 2a red labels left panel, Supplementary Figs. 3–4), and the rest were present at very low levels (0.1–10 DESeq2 normalized counts on average per tissue) (Fig. 2a orange and green labels left panel, Supplementary Figs. 3–4). Notably, palp tissue showed a very different expression pattern: only 31 out of 159 putative ORs were found from medium to high abundances, 56 were not detected, and 72 were present at very low levels (61 of them from 0.1 to 5 DESeq2 normalized counts on average per tissue) (Fig. 2a right panel, Supplementary Figs. 3 and 5). SameOR63 was detected only in the palps, suggesting this receptor plays a palp specific role.

The most abundant putative receptor in both tissue types and in all samples was SameOrco (Fig. 2a,b), consistent with Orco’s role as a coreceptor in all ORNs expressing an OR^17^. The 10 most expressed putative ORs varied depending on the tissue type: on the antenna, SameORs 1–10 were the most expressed, from ~ 20 to 1% relative to Orco abundance (Fig. 2b, left panel). For the palp, SameORs 2, 4, 10, 21, 24, 32, 36, 49, 63, and 153 were the most present, from ~ 49 to 8% relative to Orco (Fig. 2b, right panel).

We also tested whether the 159 putative ORs in Database A are expressed differentially in tissues from female and male animals. In the antenna, we observed that only five putative ORs were differentially expressed based on sex (SameOR1, SameOR7, SameOR25, SameOR40, and SameOR152) (Fig. 2c, left panel). Of these five ORs, only the expression of SameOR40 was higher in females than in males (Fig. 2c, left panel). In the palps, two putative Ors (SameOR33 and SameOR29) were significantly more highly expressed in male than female palp tissue (Fig. 2c, right panel).

### Sensilla-specific expression of putative ORs

**Figure 3:**
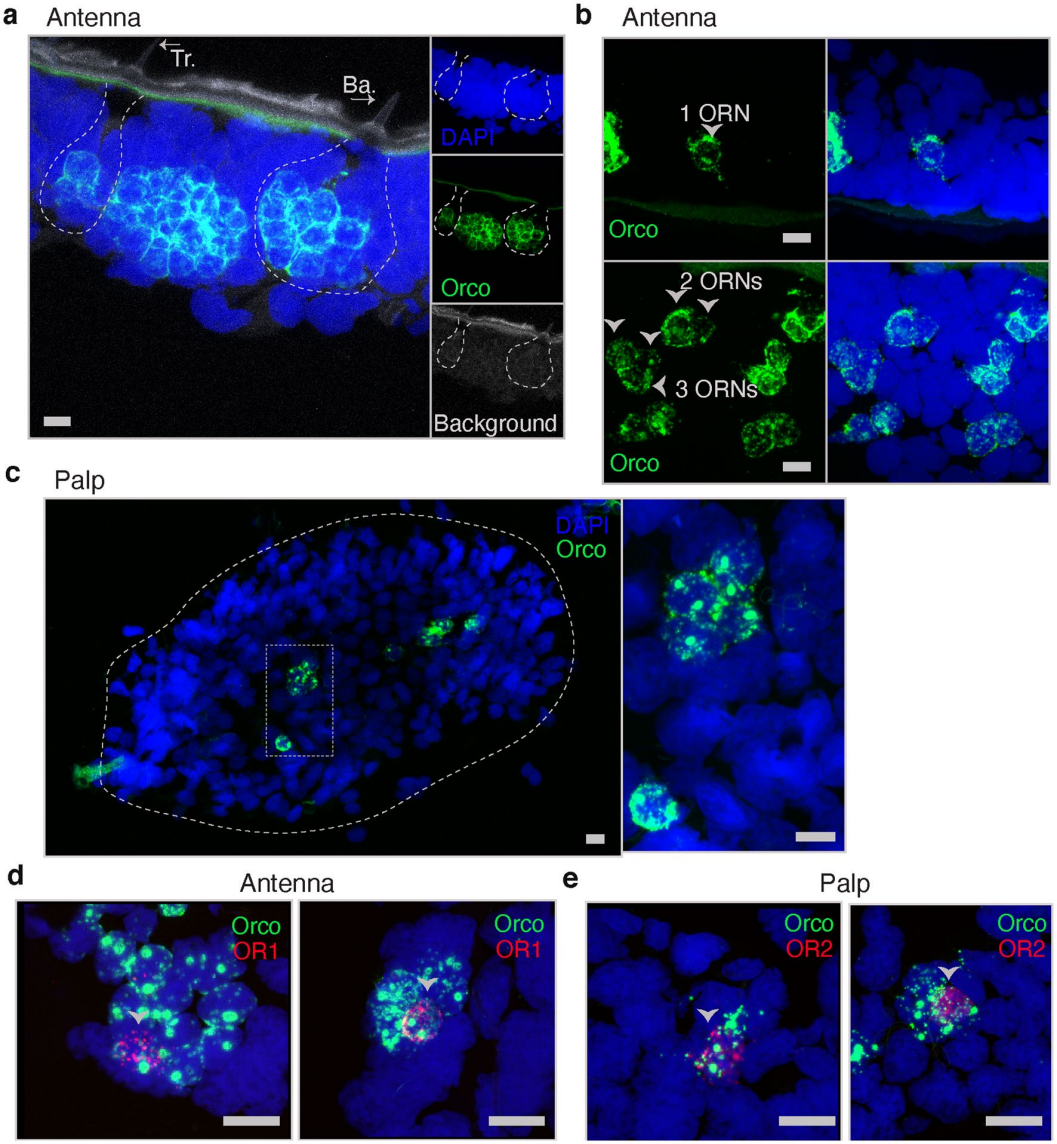
Expression of SameOrco, SameOR1, and SameOR2 in antenna and palp tissue sections, respectively. RNAscope in situ hybridization was used to characterize the spatial distribution of these putative ORs. (**a**–**c**) Probes against SameOrco (green) in antenna sections (**a**, **b**) and palp sections (**c**). Orco^+^ cells in trichoid (Tr) and basiconic (Ba) sensilla are highlighted by arrows in panel a. Trichoid sensilla containing one, two, and three ORNs are depicted by arrows in panel (**b**). (**d**, **e**) SameOrco probe with the probe against the second most expressed putative SameOR in each tissue type were used. SameOR1 (antenna, panel **d**) and SameOR2 (palp, panel **e**) are present only in Orco^+^ cells. Blue label in all images:DAPI. Scale bars: 10 μm in panels **a**–**c**; 15 μm in panels **d**–**e**.

In insects, ORNs are housed in olfactory sensilla. A subset of these neurons expresses ORs, all of which are accompanied by the OR co-receptor Orco. Normally, a single ORN expresses Orco together with only one type of OR^15,17–19^. To characterize the expression patterns of putative OR sequences generated by our de novo assembly, we next used RNAscope to perform in situ hybridization in longitudinal sections of adult antenna. In two species closely related to *S. americana*, *S. gregaria* and *L. migratoria*, Orco is expressed only in ORNs in trichoid and basiconic sensilla^37^. Here, as expected, RNAscope revealed highly abundant SameOrco expression in clusters of cells along the antenna (Fig. 3a,b). The expression patterns of SameOrco allowed us to identify trichoid and basiconic sensilla based on the number of SameOrco-expressing somata in each of the clusters: trichoid were identified as containing very few ORNs (from one to three ORNs) and basiconic, many more (Fig. 3a,b)^21^. Further, clusters of SameOrco^+^ cells were observed in sections of palp tissue but with fewer cells than in the antenna samples (Fig. 3c). These results establish that SameOrco is expressed in patterns expected for the co-receptor Orco*.*

In addition to characterizing SameOrco expression, we determined the spatial distributions of the second two most-expressed putative ORs in each of the tissues (Fig. 2). Using SameOrco to identify ORNs, we tested SameOR2 in the palp, and SameOR1 in the antenna. The results showed expression of these putative ORs exclusively in Orco^+^ cells in multiple sensilla in each analyzed section, and only in basiconic sensilla (Fig. 3d,e). Also, in some cases we found expression in multiple ORNs within the same cluster of Orco^+^ cells (Supplementary Fig. 6). These expression patterns are consistent with the initial transcript annotations as ORs.

**Figure 4:**
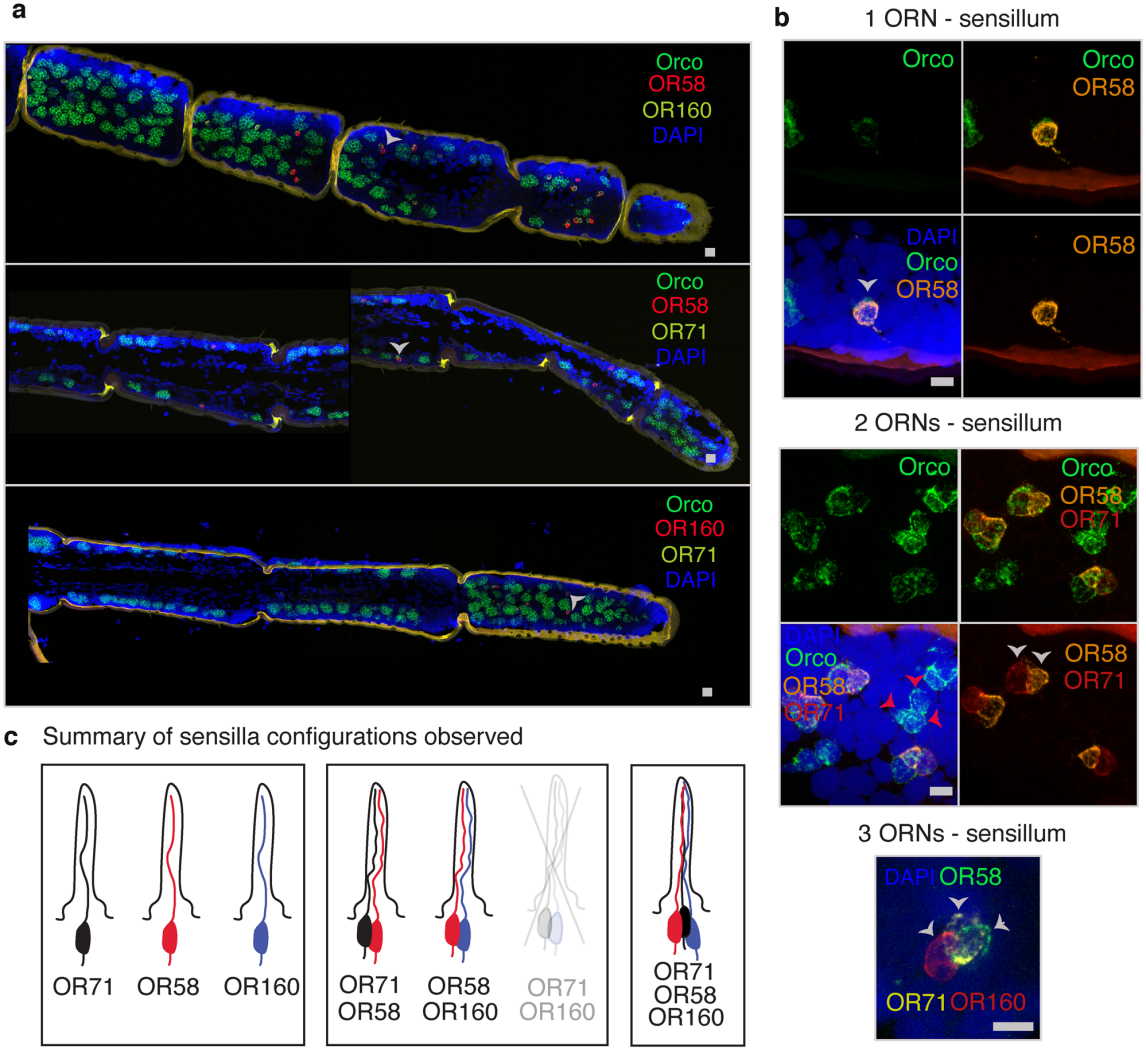
Characterization of SameOR71, SameOR58, and SameOR160 expression in trichoid sensilla. RNAscope in situ hybridization was used to determine the spatial distribution of these putative ORs. (**a**) Examples of antenna sections showing different combinations of probes against SameOrco and SameOR58 + SameOR160 (top), SameOR58 + SameOR71 (middle), and SameOR160 + SameOR71 (bottom), respectively. (**b**) Examples of individual trichoid sensilla containing one, two, or three ORNs and different combinations of the SameORs tested and indicated by gray arrows. Note that the images showing one and two ORNs are the same as in Fig. 3b but with additional color channels. Middle panel: red arrows: a cluster of three Orco^+^ cells that do not express either of the two tested putative SameORs. (**c**) Summary of the configurations observed in trichoid sensilla. Scale bars: 20 and 10 μm in panel a and b, respectively.

Sensilla organization at the molecular level is thought to play important roles in olfactory coding^5^. Individual ORNs express only one OR type in addition to Orco^19^, however whether multiple ORNs housed in the same sensillum express the same or different OR types is unknown for *S. americana*. To start an analysis of this organization in the locust we focused on trichoid sensilla in the antenna because they contain relatively few ORNs, making the analysis tractable. We tested three putative ORs: SameOR58, SameOR71, and SameOR160. These candidates were selected because of their homologies to putative ORs previously described as present only in trichoid sensilla in the desert locust (SgreOR3–SameOR58 97% identity, SgreOR102–SameOR160 97% identity, and SgreOR111–SameOR71 98% identity)^38^. We found that, as in the desert locust, these putative receptors were expressed in SameOrco^+^ ORNs in trichoid sensilla (Fig. 4a–c). However, in rare instances we also found SameOR160 expressed in ORNs in basiconic sensilla (only two cases among hundreds of examined sensilla; Supplementary Fig. 7).

After confirming that these three SameORs were expressed in trichoid sensilla, we characterized their structural and molecular organization with an in situ RNAscope analysis of their expression patterns. Trichoid sensilla can contain one, two, or three ORNs. We used in situ RNAscope for SameOrco plus SameOR58, SameOR71, and SameOR160 to assess which combinations were present. Unlike the case of SameOR1 or SameOR2 that were found in one or more ORNs within the same basiconic sensillum, none of the trichoid sensilla we examined (of ~ 400–600 sensilla per condition across animals) contained more than one ORN expressing the same OR type (SameOR58, SameOR71, or SameOR160). In addition, we regularly observed the following combinations: sensilla housing one ORN expressed SameOR58, or SameOR71, or SameOR160; sensilla housing two ORNs expressed SameOR58 and SameOR71 or, SameOR58 and SameOR160; sensilla housing three ORNs expressed SameOR58, SameOR71, and SameOR160. However, we never observed the co-expression of SameOR160 and SameOR71 in a sensillum housing two ORNs. Further, we found sensilla containing three ORNs (Orco^+^ cells) that did not express any of the three tested ORs (Fig. 4b, red arrows), indicating that ORs we did not test with RNAscope are also expressed in trichoid sensilla. Together, these results suggest the organization of ORs within sensilla is not random and likely reflects an underlying logic.

### Phylogenetic comparison of three orthopterans’ putative ORs

**Figure 5:**
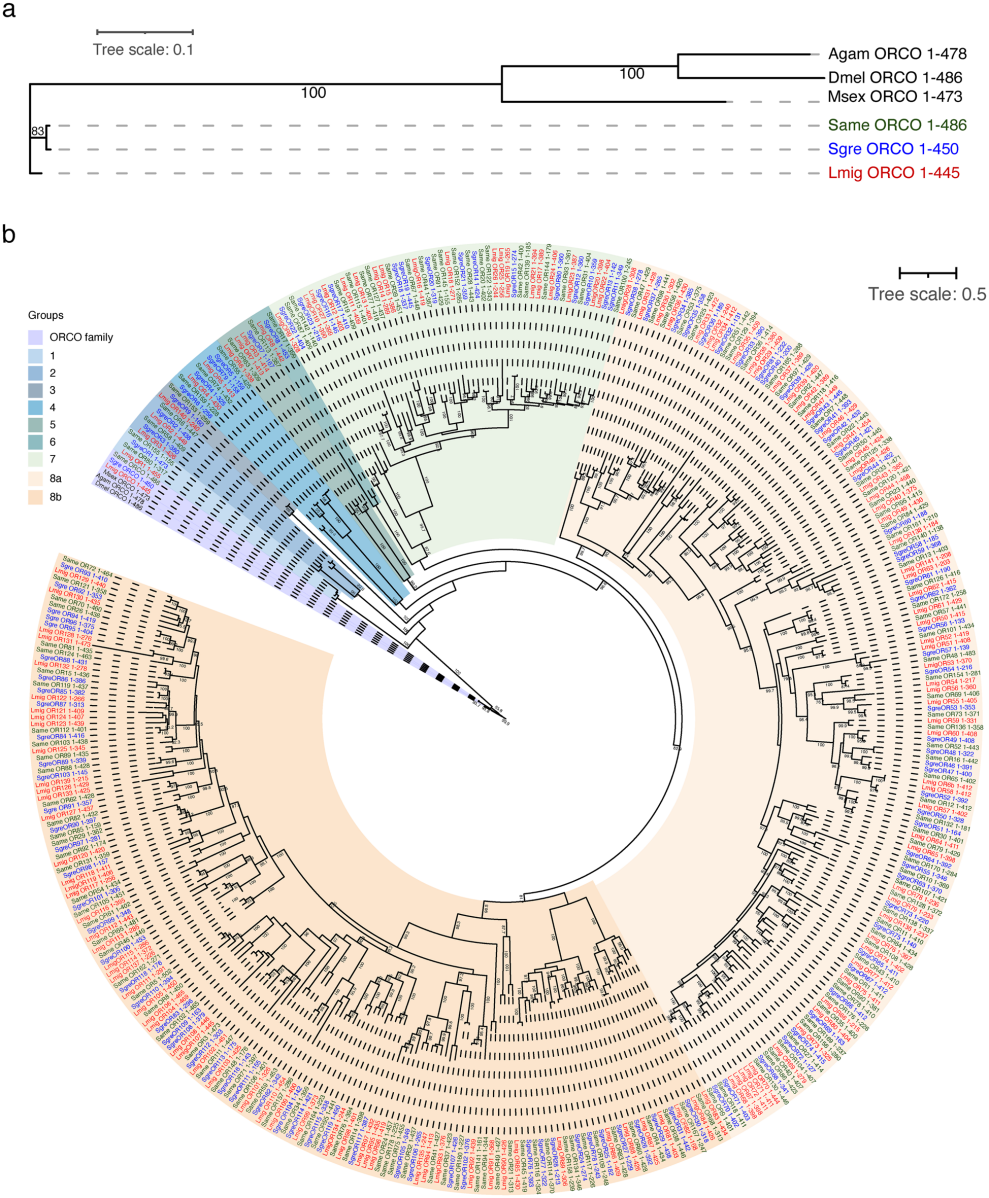
OR protein family phylogeny including putative SameORs and two other members of the *Acaridae* family (*S. gregaria*—Sgre and *L. migratoria*—Lmig). The maximum likelihood tree was generated from amino acid sequences deduced from the 159 putative ORs contained in Database A, and published ORs from *S. gregaria* and *L. migratoria* (see Methods). (**a**) Enlarged view of the co-receptor Orco subgroup shown in panel (**b**). Confidence bootstrapping values are indicated. (**b**) The tree was rooted using Orco protein family members, including *D. melanogaster* (Dmel), *A. gambiae* (Agam), *M. sexta* (Msex), Sgre, Lmig, and putative SameOrco (shown in panel **a**) protein’s amino acid sequences. Confidence bootstrapping values above 80 are shown (1000 iterations and a correlation coefficient of 0.99 were used). For descriptive purposes the ORs were divided into eight groups based on the first seven nodes. The length of each predicted protein’s amino acid sequence is included in the labels.

To further characterize and identify homologous sequences of our set of putative SameORs transcripts in other orthopterans, we performed a phylogenetic analysis. Using the 159 amino acid sequences deduced from the sequences in Database A, we generated a maximum likelihood-based phylogenetic tree by aligning them against previously reported OR sequences from two other members of the *Acrididae* family (Orthoptera order): *L. migratoria* and *S. gregaria*^39,40^. The phylogenetic tree was rooted in the highly conserved Orco protein (Fig. 5a) and included Orco proteins from the two orthopterans and insects from different orders: *D. melanogaster* (Dme), *A. gambiae* (Agam), *M. sexta* (Mse), *S. gregaria* (Sgre) and *L. migratoria* (Lmig). Putative SameOrco exhibited the characteristic sequence conservation of Orco proteins as it shared above 60% sequence identity with Orco orthologs spanning the different insect orders^41^. The highest similarity of putative SameOrco was with those from the two orthopterans Lmig and Sgre—96.63 and 99.11% identity respectively; while it shared ~ 60–61% identity with the Dme, Agam and Mse Orcos. These results support our identification of SameOrco as encoding the Orco protein. Further, we found homologous sequences for almost all putative SameORs in either of the two-orthopteran species’ ORs included in our analysis (Fig. 5b). Putative ORs in *S. americana* and *S. gregaria* were mapped more closely together in our phylogenetic analysis than those in *L. migratoria*, as shown in Fig. 5—most of them sharing > 80% sequence identity—consistent with their closer evolutionary relationship. Together, these results provide additional evidence that the transcripts we identified as putative SameORs represent genuine ORs sequences.

Previous phylogenetic analysis using of either LmigORs, or LmigORs against SgreORs^39,40^ have shown clusters of ORs forming multiple families including two major groups with larger expansions. Following a similar methodological approach, we observed that the putative SameORs together with Sgre and Lmig ORs form similar phylogenetical clusters supported by high confidence values (> 80), as previously observed^39,40^. Based on the first seven nodes present in the tree, we divided the ORs into eight groups. Groups 1–6 radiated relatively early from the Orco-family and contain 11 putative SameORs. The small number of ORs present in Groups 1–6 represent relatively little evolutionary expansion with only one or two *Acrididae* OR members per clade. The remaining OR sequences were mapped into Groups 7 and 8, more distant from the Orco-family groups, as proposed previously^39,40^. Group 7 contains 21 putative SameORs, while group 8 contains 126, with 63 and 63 in each of its two subgroups (8a and 8b respectively). These results support earlier observations that the *Acrididae* OR superfamily expanded from a common ancestor, which, after some possible initial duplications, gave rise to a set of groups with different extents of expansion. Our phylogenetic results, together with the previous reports from *L. migratoria* and *S. gregaria*, help characterize the evolution and conservation of the *Acrididae* OR protein superfamily. However, further genome sequencing of organisms within this family, together with complementary genetic and functional studies, will shed light on the details of its evolutionary history and functional relationships.

## Discussion

In insects, olfaction begins when odorants bind with odor-sensitive proteins including ORs, ionotropic receptors (Irs), and some gustatory receptors^42^. Here, we identified a total of 179 transcripts of putative ORs in the antenna and palps, the two main olfactory organs of *S. americana,* and further characterized 159 putative ORs in terms of their differential expression patterns in the antennae and palps of female and male locusts. We also examined their phylogenetic relationships with closely related species, and homologies between SameORs to ORs of *S. gregaria* and *L. migratoria*. We also determined the spatial locations of a subset of these putative ORs within different types of sensilla, and, based upon their expression patterns, obtained evidence suggesting the organization of ORs within sensilla is not random but rather reflects an underlying logic. We also identified a single putative OR (SameOR63) expressed in the palps but not the antennae. Together, these results provide a new and precise view of OR expression in the locust, and useful tools for further exploration of an animal that is both a valuable model system for studies of sensory processing, and of economic interest since it can represent an agricultural pest.

### Assembly and identification of putative SameORs

We used a de novo method using the Trinity platform to assemble the transcriptome of antennal and palp tissues (see “Methods”). De novo methods to assemble RNAseq data do not require the use of a genome for reference and are widely used^36,43^. When it became available during our work, we then used the recently posted genome assembly of *S. americana* (GenBank GCA_021461395.2) as an additional tool to validate our approach and to help annotate the putative ORs we had identified.

Our high quality sequencing and assembly allowed us to identify 179 putative ORs in *S. americana,* more than had been reported in other orthopterans including the 142 and 119 putative ORs previously identified in *L. migratoria*^39^ and *S. gregaria*^40^, respectively. Our search for ORs included data pooled from antenna and palp transcriptomes to create a unified assembly, while the other studies combined antenna transcriptomes with locust genome data^39^, or used the antenna transcriptome only^40^. The latter approach would exclude putative ORs that are palp specific—for example, SameOR63. Our study also used relatively deep sequencing to identify potentially low-abundance transcripts; we generated 1.4724 × 10^9^ paired end reads, compared to 9.5674882 × 10^7^ reads generated in *L. migratoria*^39^ and 5.1151235 × 10^7^ paired end reads generated in *S. gregaria*^40^. Our annotation process also benefited from comparisons to published putative OR sequences from *L. migratoria* and *S. gregaria* together with putative ORs from other members of *Acrididae* family (see Methods).

Insects ORs, other than Orco, are highly divergent with little sequence homology within insect species and, in some cases, even across species of the same order^44^, complicating the process of identifying and annotating candidate OR-encoding sequences. Our sequencing and assembly methods revealed 107 sequences had full-length coding regions, equivalent to 59.7% of the full set of 179, more than previously reported for related species: 54% (77 putative ORs) and 15% (18 putative ORs) in *migratoria*^39^ and *gregaria*^40^, respectively. Moreover, the majority of the protein sequences of the putative OR CDSs we identified (64.2%) had predicted structural topologies containing at least six TMDs, as expected for OR proteins. It is possible that shorter sequences in our dataset with incomplete CDSs are poorly seeded assembly fragments (*i.e.,* noise) derived from the assembler, pseudogenes, or even pseudo-pseudo genes as is the case for some OR genes in *Drosophila melanogaster*^45^. We divided our full set of putative SameORs into two databases. Database A includes sequences most likely to encode genuine SameOR proteins: those with multiple predicted TMDs^13–16^ and high homologies with those of closely related species, as revealed by our phylogenetic analysis. We limited our expression and phylogenetic analyses to Database A. Database B includes shorter sequences—some possibly sharing identities with putative SameORs contained in Database A—and some lacking multiple predicted TMDs. We provide Database B because future analyses may reveal it also contains some genuine ORs. Functional studies are now needed to determine whether these sequences encode fully functional ORs.

### Tissue specific expression of putative SameORs

*S. americana*, like other insects, has two main olfactory organs: the antennae and the palps. We found these two organs expressed different but mainly overlapping sets of putative ORs. First, the majority (141 of 159) of the putative ORs were present in the antenna while less than a fifth of that number (only 31) were observed in the palp in moderate to high abundance. This difference is not surprising given that the palps contain three orders of magnitude fewer ORNs than the antenna (~ 200 vs ~ 100,000^25–29^ and each ORN expresses only one type of OR together with Orco. Similar organ-specific differences have been observed in other insects. For example, in *L. migratoria*, of 149 putative OR genes identified, only 11 have been found in the palps^46^; in *A. gambiae*, of ~ 80 putative ORs, only 13 are in the palps^47^; in *D. melanogaster*, of 62 ORs, only seven are present in the palps^48^; in *M. sexta*, of 70 OR genes tested, 17 are found in the mouthparts^49^.

Second, we found that all but one of the putative ORs identified in the palps were also observed in the antenna. This overlap is different from the case of *Drosophila,* in which ORs expressed in antenna and palp are mutually exclusive^48^. However, observations from other species including the dipteran *A. gambiae*^47^, the lepidopteran *M. sexta*^49^, the orthopterans *L. migratoria*^46^ and *S. gregaria*^50^, and our results, show substantial overlap of OR types expressed in both structures, suggesting that OR mutually exclusive expression of ORs in antenna and palp tissues of *Drosophila* appears to be exceptional.

This partitioning of OR types to distinct olfactory structures may in some ways be comparable to the mammalian olfactory system, in which the molecular identities of ORNs in the main olfactory epithelium and the vomeronasal organ also overlap to some degree^51^. In mammals, ORNs from these two peripheral olfactory areas project to different target regions in the brain: ORNs from the main olfactory epithelium project into the main olfactory bulb, while the axons from vomeronasal ORNs expressing the same OR type project to the accessory olfactory bulb^51^. Similarly, in locust, antennal ORNs project to the antennal lobe, but palp ORNs project to the lobus glomerulatus^29^. Functional studies are needed to determine whether odor information traveling through these pathways is processed differently or plays distinct roles.

Only one of the putative ORs we identified—SameOR63—was expressed exclusively in the palps, raising the possibility that it plays a specialized role in feeding. A homologue for SameOR63 was not identified in *S. gregaria*, but in *L. migratoria*, LmigOR6^39^—also known as LmigOR12^46^—shares 76.3% identity with SameOR63. A previous report showed that LmigOR12 was also found in the palps but not in the antennae of adult animals^46^. Prior electrophysiological studies in *L. migratoria* have shown that certain odorants (e.g., (E,E)-2,4-heptadienal, hexanal and E-2-hexenal) elicited stronger responses in the palps than in the antennae, and that these responses were diminished when LmigOR12 expression was knocked down via RNAi^46^. Several other putative ORs we identified in *S. americana,* in addition to SameOR63, are differentially expressed in the antennae and palps. These results at the molecular level between these two olfactory organs may reflect differences in their functions.

### Sex differences in putative SameOR expression

In holometabolous insects (those undergoing complete metamorphosis during development) it is common to observe sexual dimorphic expression of some ORs. In Diptera^47,52^ and Lepidoptera^53–55^, for example, receptors that detect pheromones are most often expressed differentially in females and males. However, less is known about pheromone detection or differences in OR expression based on sex in hemimetabolous insects, including orthopterans like the locust. Furthermore, there are no obvious differences between female and male antennal sensilla arrays in orthopterans *S. gregaria*^21^, *L. migratoria*^56^, or *S. americana*^57^, suggesting that, if olfactory sexual dimorphism exists, it would be present only at the molecular level. In agreement with this hypothesis, our results showed that, in *S. americana*, at least five putative ORs in the antenna and two in the palps were differentially expressed by sex. A previous report in *S. gregaria*^50^ did not find evidence for sexual differences in the expression of putative ORs in either antennae or palps. However, the evidence provided for *S. gregaria* may not be sufficient to rule out the possibility of sex differences in the expression of OR proteins, since only nine putative ORs were analyzed in the study^50^. Thus, the apparent discrepancy between our results and those from *S. gregaria* could be explained because none of the putative ORs we found to be differentially expressed were analyzed in *S. gregaria*^50^. Whether these putative ORs detect pheromones remains to be tested.

Further, some pheromone receptors have already been identified in locust^40,50,58^. For example, in *L. migratoria,* the aggregation pheromone 4-vinylanisole (4VA) was shown to be detected by LmigOR35^58^; knocking down this receptor impaired behavioral responses normally elicited by this odorant. In our database, SameOR36, which is expressed on antennae and palps, comes closest to matching LmigOR35 (75% identity) and would be a good candidate for future analysis.

### Organization of SameORs in sensilla

ORNs are housed in olfactory sensilla. Three types of sensilla, basiconic, trichoid and coeloconic, are found in the antenna, but only basiconic sensilla have been observed in the palps^25,26^. In locust, basiconic and trichoid sensilla contain ORNs that co-express ORs with Orco. In *Drosophila* as well as some other species, sensillar organization in the antenna and the palps at the molecular and functional levels has been shown to be stereotypical^5^. However, in the locust this organization is unknown. The molecular organization of sensilla likely has functional consequences because ephaptic communication—nonsynaptic neural interactions—among ORNs closely apposed within a sensillum can influence their mutual responses to odors^22^. Therefore, it is important to understand sensillar organization as a contributor to early stages of odor coding.

To make our initial analysis of the organization of ORs into sensilla tractable, we focused mainly on trichoid sensilla, which each house only one to three ORNs. We identified trichoid sensilla as those containing small numbers of SameOrco expressing ORNs, and then analyzed them based on the patterns of putative ORs expressed by their ORNs. Our analysis confirmed the expression of at least three putative OR types in these sensilla. These results are consistent with a study performed in the trichoid sensilla of *S. gregaria* that identified the expression of ORs homologous to the three we tested^40^, and to another study performed in *L. migratoria* that identified the expression of an OR homologous to SameOR58^59^, suggesting the conservation of the spatial organization of these ORs as orthopterans evolved. Further, we observed that some combinations of these ORs were often expressed together in a sensillum, but other combinations were never expressed together. Our sample of three ORs is too small to draw specific conclusions, but we broadly interpret these results to suggest the organization of ORs within sensilla is not random and likely reflects an underlying logic^22^. An exciting direction for future work will be to explore this logic by examining more ORs and their functions.

Finally, we found evidence that additional combinations of ORs within trichoid sensilla are present in the antenna. For example, we observed cases in which none of the three Orco^+^ neurons housed in a trichoid sensillum expressed any of the putative ORs tested. Each type of OR appears to be expressed in only one ORN per trichoid sensillum. Because we tested at least two different putative ORs in each experiment, it is likely that at least two other OR types that were not tested by RNAscope in this study are also expressed in trichoid sensilla. In addition, our observation that at least one of the putative ORs present in trichoid sensilla is also expressed in basiconic sensilla points to the possibility that additional OR types could be present in trichoid sensilla. However, the co-expression of this OR in both trichoid and basiconic sensilla was unusual because we observed it only once. In *L. migratoria*, 16 functional classes of trichoid sensilla have been described^60^ which could be achieved by the presence of ~ five OR types.

Basiconic sensilla outnumber others on the locust antennae (< 2000, compared to ~ 500 trichoid sensilla), each contain many ORNs (20–50, compared to one to three in trichoid sensilla), and are the only sensillar type found in the palps^21,26–28,57^. Thus, one might expect the great majority of ORs to be expressed within basiconic sensilla. It will be important to explore the functional organization of basiconic sensilla in future studies.

## Methods

### Animal rearing

*Schistocerca americana* used in this study were raised in our large, crowded colony, in screened cages (45 × 45 × 45 cm) at a density of 300–400 per cage. The colony was maintained in a 12 h light 12 h dark cycle at ~ 30 °C and fed fresh wheat grass and oat bran.

### RNA extraction and sequencing

We performed RNA sequencing to compare the molecular expression profiles of antenna and palp tissues in male and female adult *S. americana*. Three biological replicates were analyzed per condition, each consisting of left and right antennae or a mix of maxillary and labial palps from 5 adult females or males. Samples were collected and stored in RNAlater reagent (Qiagen) to prevent degradation, and then total RNA was extracted from the samples using Rneasy plus mini kit (Qiagen) and gDNA eliminator columns in accordance with the manufacturer’s protocol. RNA samples were then assessed for concentration, purity, and integrity using Nanodrop and Bioanalyzer. Only samples with high purity and minimal degradation (absorbance ratio at 260/280 of ~ 2.0 and RNA integrity number—RIN—above 8) were used for subsequent steps. cDNA library preparation and sequencing were performed in the Molecular Genomics Core (NICHD-NIH, Bethesda MD). To prepare libraries, 1 to 3 μg of RNA was used and mature poly-A transcripts were enriched using a TruSeq RNA library preparation kit (Illumina). Subsequently, samples were sequenced using Illumina HiSeq 2500 and NovaSeq 6000 platforms (100 bp paired end reads). A total of 1,472.4 million clean (*i.e.,* post-trimmed) read pairs were obtained from all 12 tissue samples with a mapped ratio, on average, of 83% (Supplementary Table 1).

### Assembly of RNA sequences

Before assembly, data were cleaned and trimmed using cutadapt software v2.5 (–a AGATCGGAAGAGCACACGTCTGAACTCCAGTCA -A AGATCGGAAGAGCGTCGTGTAGGGAAAGAGTGT—overlap 6 − q 20—minimum-length 25). Sequences generated were assembled in Trinity v2.12.0 using a de novo assembly pipeline as previously described^61^. Briefly, reads from both tissue types were pooled and passed into the processing pipeline to create a unified de novo transcriptome assembly with software default parameters using 32 CPUs and 350 Gb of memory. This process generated a total of 850,291 contigs of which 60,863 contained ORFs (Supplementary Table 2). The completeness of the assembly was assessed with the TransRate assembly score and the benchmarking sets of universal single-copy orthologs (BUSCO) v3.0.2 completeness assessment tool (Supplementary Table 2, Supplementary Fig. 1).

### OR annotation

To identify putative ORs sequences, contigs were analyzed with BLASTn searches using Blast v2.13.0 software against a custom database containing annotated putative ORs from several related species, *Schistocerca gregaria, Locusta migratoria, Ceracris nigricornis*, and *Oedaleus infernalis* (Supplementary Fasta 1) and all labeled odorant receptors from the National Center for Biotechnology Information (NCBI) nucleotide database (Supplementary Fasta 2). From the extracted transcripts, we predicted all possible ORFs using TransDecoder software and analyzed with protein BLASTp searches against the NCBI and UniProtKB/Swiss-Prot databases. Only those sequences producing e-values below 1E^-7^ were defined as hits. This procedure allowed us to identify chimeric assembly transcripts and extract regions of the transcript that aligned to OR sequences, and to confirm that the predicted coding sequences (CDSs) matched to only ORs and not to a non-OR protein. Predicted CDSs aligning only to an OR were extracted, and those > 300 bp in length were defined as putative SameOR sequences.

To help validate our approach, we performed a BLASTx search of our putative OR sequences against a genome assembly for *S. americana* recently deposited in NCBI (GenBank GCA_021461395.2) and identified their loci (Supplementary Data 1). Only those sequences producing e-value scores below 1E^-7^ were considered hits.

To increase the likelihood that the extracted sequences encoded ORs, we applied two additional analyses. First, because ORs are expected to have multiple TMDs^13–16^, we used deepTMHMM software^62^ to check for them in the amino acid sequences predicted from the CDSs (Supplementary Fasta 3 for CDSs and 4 for the predicted protein sequences). Second, we aligned the putative SameOR derived amino acid sequences using the clustal Omega Multiple Sequence Alignment web tool (https://www.ebi.ac.uk/Tools/msa/clustalo/) and checked for predicted amino acid sequence lengths and redundancy. Based on these results we collected those non-redundant (i.e., a shared identity above 90%) transcripts with two or more TMDs and lengths above 400 bp (~ 133 amino acid sequence length) in Database A (Supplementary Data 1), and the rest of the putative ORs in Database B (Supplementary Data 2). In addition, we determined the completeness of the CDSs by checking for the presence of start and stop codons using TransDecoder (Supplemental Data 1 and 2).

### Reverse transcription polymerase chain reaction (RT-PCR)

To further validate our putative ORs, we checked for their presence in the antennae and palps of female and male locusts. Briefly, total RNA was extracted from antennal and palp tissue following the protocol described above. Using Oligo-dT primers and the GoScript Reverse Transcription System (Promega) the first strand of cDNA was synthesized from 1 μg of RNA in a reaction volume of 20 μl. Synthesis was conducted at 42 °C for 60 min and was followed by 15 min incubation at 70 °C. Gene-specific primers were designed with Primer-BLAST (Supplementary Table 3) spanning an exon junction when possible. In addition, a negative control condition without reverse transcription for each sample was processed for Orco amplification which allowed us to rule out the possibility of genomic DNA contamination. PCRs were performed using GoTaq Green Master Mix (Promega). For all samples, 1 μl of cDNA was used as template in a reaction volume of 25 μl with the following conditions: 95 °C for 2 min, followed by 35 cycles with 95 °C for 30 s, 65 °C for 40 s, and 72 °C for 20 s, and, after the last cycle, a final incubation at 72 °C for 5 min. All PCR products in a given experiment were run in the same gel (2% agarose) and visualized by staining with SYBR green. Images of the gels were acquired with the SmartDoc Imaging System for smartphones and processed in Image J (i.e., 8-bit image conversion, rotation, and LUT inversion). The lengths of the amplified PCR products were 150–764 bp. PCR products were purified (Nucleospin gel and PCR clean-up, Macherey–Nagel) and sequenced using Sanger for verification (Eurofins Genomics).

### Phylogenetic analysis

A phylogenetic analysis of *S. americana* putative ORs was performed using the 159 sequences contained in Database A. We used the 159 aligned amino acid sequences of SameOR against the previously identified *S. gregaria* and *L. migratoria* OR amino acid sequences^39,40^ (Supplementary Data 3 and 4). Alignments obtained from CLUSTALO were used to generate a maximum likelihood-based tree with the IQ-Tree software package (http://www.iqtree.org)^63^ using the “auto” substitution model and default values for Perturbation Strength and IQ-tree stopping rule. The tree was generated using the ultrafast bootstrap approximation approach (UFBoot2)^64^ with 1000 iterations and a 0.99 correlation coefficient. The phylogenetic tree was rooted using the Odorant Receptor CO-receptor (Orco) sequences of five insect species (*D. melanogaster, A. gambiae, M. sexta, S. gregaria, L. migratoria*) and the deduced amino acid sequence of the *S. americana* Orco identified in this work. Also, these six Orco sequences were aligned with CLUSTALO (Supplementary Data 3) and a maximum likelihood-based tree was generated with the IQ-tree software using the same parameters previously described for the OR sequences. iTOL (https://itol.embl.de/)^65^ was used to visualize and annotate the dendrograms.

### Differential expression analysis

Differential expression analysis of predicted putative ORs was performed by aligning trimmed RNAseq reads against the indexed full putative OR CDS subset using kallisto v0.46.0^66^. The resulting quantification was imported into DESeq2 for differential expression testing^67^ across separate female and male tissue samples using tximport as described in DESeq2 vignettes using a two-factor tissue and gender model. Gene expression in female and male tissue was considered significantly different when characterized by an adjusted p-value (padj) of under 0.1.

### RNAscope multiplex fluorescent assay

Fresh frozen sections of antenna and maxillary palp tissues were processed following a standard protocol (Advance Cell Diagnostics Inc., Newark, CA). Briefly, antennal distal segments 1–14 and maxillary palp dome were cut in longitudinal and coronal serial sections, respectively, of 14–16 μm. These regions were selected because they contain most olfactory sensilla in the adult: the antenna segments 1–14 contain > 80% of basiconic and trichoid sensilla^57^ while the palp’s dome contains all of the basiconic sensilla^25,26,31^. Serial sections were mounted onto SuperFrost plus slides, dried at room temperature, and stored in air-tight slide boxes at − 80 °C until further processing. Sections were fixed with cold 4% PFA for 15 min at 4 °C followed by ethanol dehydration. For pre-treatment, slides were incubated with RNAscope Protease III for ~ 25 min at 40 °C using a HybEZ oven. Slides were incubated with customized probes for SameOrco (871191-C1) plus one or a combination of the following: SameOR1 (1201441-C2), SameOR2 (1201451-C3), SameOR58 (previously named SameOR3, 871201-C2,), SameOR71 (previously named SameOR111, 872061-C4), SameOR160 (SameOR102, 871221-C3) for 2 h at 40 °C, followed by signal amplification and detection using the RNAscope Multiplex Fluorescent Reagent Kit version 2. For positive and negative control conditions we used a customized probe for SameGAPDH (872871) and DapB (of *Bacillus subtilis* strain) provided by the manufacturer (321831). For signal detection, Opal 520, 570, 620, and 690 fluorophores (AKOYA Biosciences, Marlborough, MA) were prepared at 1:1000 dilution in TSA buffer. Sections were counterstained with DAPI and mounted with ProLong Diamond Antifade Mountant (Thermo Fisher Scientific). Samples were visualized and image were acquired with a Zeiss LSM 880 Airy confocal microscope. Each condition was replicated in at least two animals. The number of sections analyzed per antenna segment for each condition ranged from 3 to 6 per animal. All confocal images shown are maximum projections unless otherwise stated.

## Supplementary Information

Supplementary Information 1.

Dataset S1.

Dataset S2.

Dataset S3.

Dataset S4.

Supplementary Information 2.

Supplementary Information 3.

Supplementary Information 4.

Supplementary Information 5.

## Data Availability

The data generated and analyzed during the current study is available in SRA repository under the BioProject ID PRJNA889432 [https://www.ncbi.nlm.nih.gov/sra/PRJNA889432] and in GenBank repository with the accession numbers: OP777696–OP777865, OP777867, OP777871–72, OP777874, OP777877–79, OP777883, OP777892. In addition, data is included in this manuscript and its supplementary information files.
