# Supplementary material for: Identification and analysis of odorant receptors expressed in the two main olfactory organs, antennae and palps, of Schistocerca americana
Source: Sci Rep. 2022 Dec 31;12:22628. doi: 10.1038/s41598-022-27199-3 (PMC9805433; doi:10.1038/s41598-022-27199-3)
Supplement: Supplementary file 1 — Supplementary Information 1. [file 41598_2022_27199_MOESM1_ESM.docx]

**Supplementary information**

**Identification and analysis of odorant receptors expressed in the two main olfactory organs, antennae and palps, of *Schistocerca americana***

Alejandra Boronat-Garcia^1^, James Iben^2^, Eunice Dominguez-Martin^3^, Mark Stopfer^1^*

*Corresponding author: [stopferm@nih.gov](mailto:stopferm@nih.gov)

^1^Section on Sensory Coding and Neural Ensembles, National Institutes of Health, Eunice Kennedy Shriver National Institute of Child and Human Development, Bethesda MD

^2^Molecular and Genomics Core, National Institutes of Health, Eunice Kennedy Shriver National Institute of Child and Human Development, Bethesda MD

^3^Biochemestry Department, National Institutes of Health, National Institute of Neurological Disorders and Stroke, Bethesda MD

| **ID tissue sample** | **Number of read pairs (Million)** | **GC content** | **Number of clean read pairs (Million)** | **Mapped read pairs (Million)** | **Mapped ratio** |
| --- | --- | --- | --- | --- | --- |
| FA1 | 37.8 | 45% | 37.7 | 31.4 | 83% |
| FA3 | 30.7 | 45% | 30.6 | 25.5 | 83% |
| FA4 | 33.3 | 45% | 33.2 | 27.6 | 83% |
| MA2 | 34.7 | 44% | 34.6 | 28.6 | 83% |
| MA3 | 37.9 | 45% | 37.8 | 31.4 | 83% |
| MA4 | 34.7 | 44% | 34.6 | 28.6 | 83% |
| FP1 | 218.9 | 45% | 217.9 | 180.3 | 83% |
| FP3 | 208.8 | 46% | 208.1 | 173.5 | 83% |
| FP4 | 218.3 | 46% | 217.2 | 181.5 | 84% |
| MP2 | 226.6 | 45% | 226 | 190.2 | 84% |
| MP3 | 192.1 | 44% | 191.4 | 161.4 | 84% |
| MP4 | 204.1 | 45% | 203.3 | 168.8 | 83% |

**Supplementary Table 1.** RNAseq read pairs information. Three replicates per condition were analyzed. Each replicate contained right and left antenna or palp tissue pooled from 5 animals.

| **Statistic** |  |
| --- | --- |
| Number of sequences | 850,291 |
| Smallest | 171 |
| Largest | 49,174 |
| Number of bases | 617,409,033 |
| Mean length | 726 |
| Number of sequences under 200 bp | 167 |
| Number of sequences over 1kbp | 134,704 |
| Number of sequences over 10 Kbp | 1,215 |
| Number with ORF | 60,863 |
| Mean ORF (percentage) | 38.6% |
| N90 | 270 |
| N70 | 540 |
| N50 | 1,383 |
| N30 | 2,994 |
| N10 | 6,206 |
| GC ratio | 0.405 |
| TransRate Score | 0.855 |
| Percent Mapping | 83.0% |

**Supplementary Table 2**. Statistics of the *de novo* generated assembly by Trinity v2.12.0.


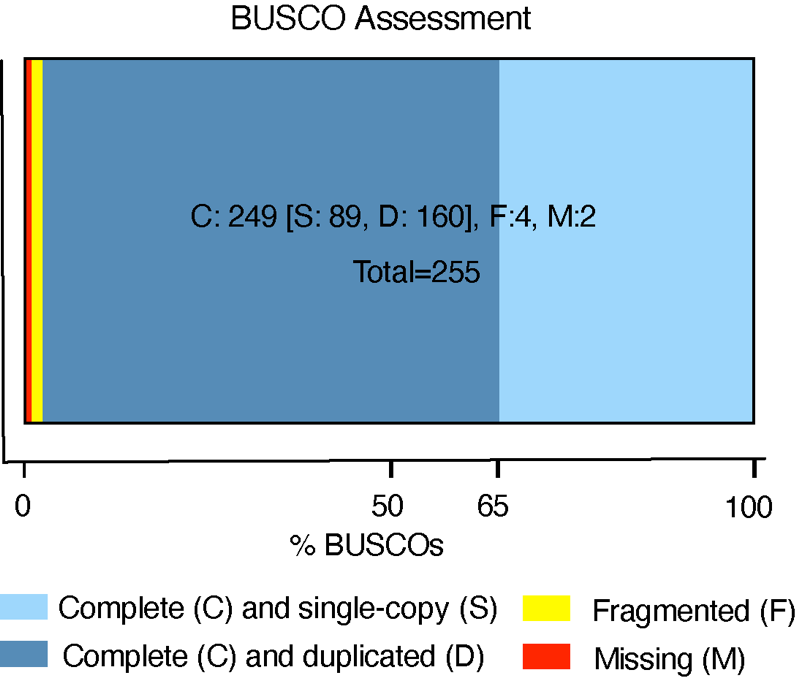


**Supplementary Figure 1.** BUSCO assessment results of antenna and palp transcriptome. Light blue: complete and single copy; dak blue: complete and duplicate; yellow: fragmented; and red: missing genes.

**
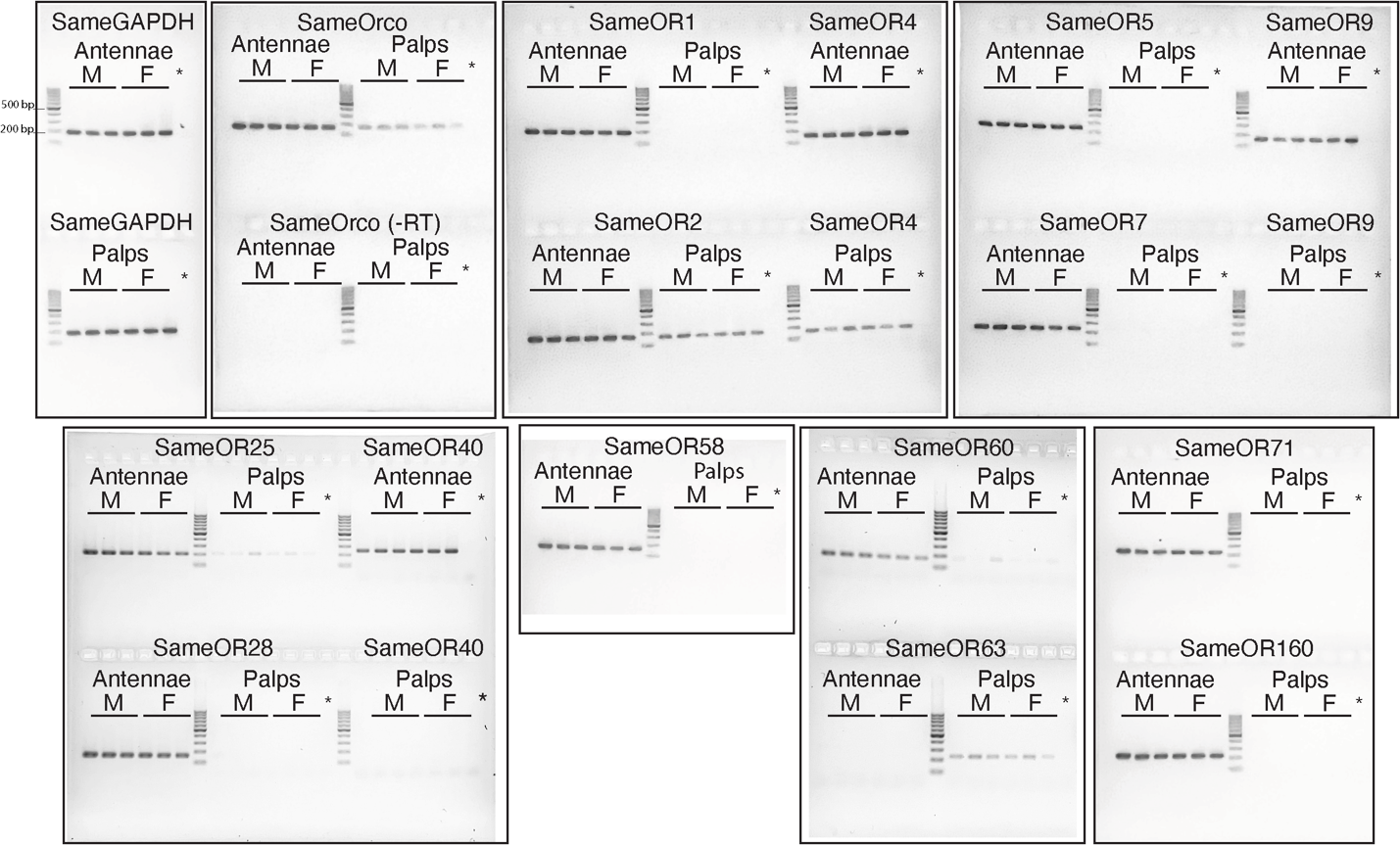
**

**Supplementary Figure 2.** RT-PCR amplification of 16 transcripts from antenna and palp tissue of male (M) and female (F) locust. SameGAPDH was used as a positive control. SameOrco without reverse transcription (-RT) was used as a negative control. Asterisks indicate a ‘no template’ sample used as an additional negative control. Equal volumes of amplified products were loaded in 2% agarose gels containing SYBR green. Images of gels were acquired with the SmartDoc Imaging System for smartphones and processed in Image J (i.e., 8-bit image conversion, rotation, and LUT inversion). For all cases, a band of the expected size was amplified, and the PCR products were purified and sequenced using Sanger for verification.

**
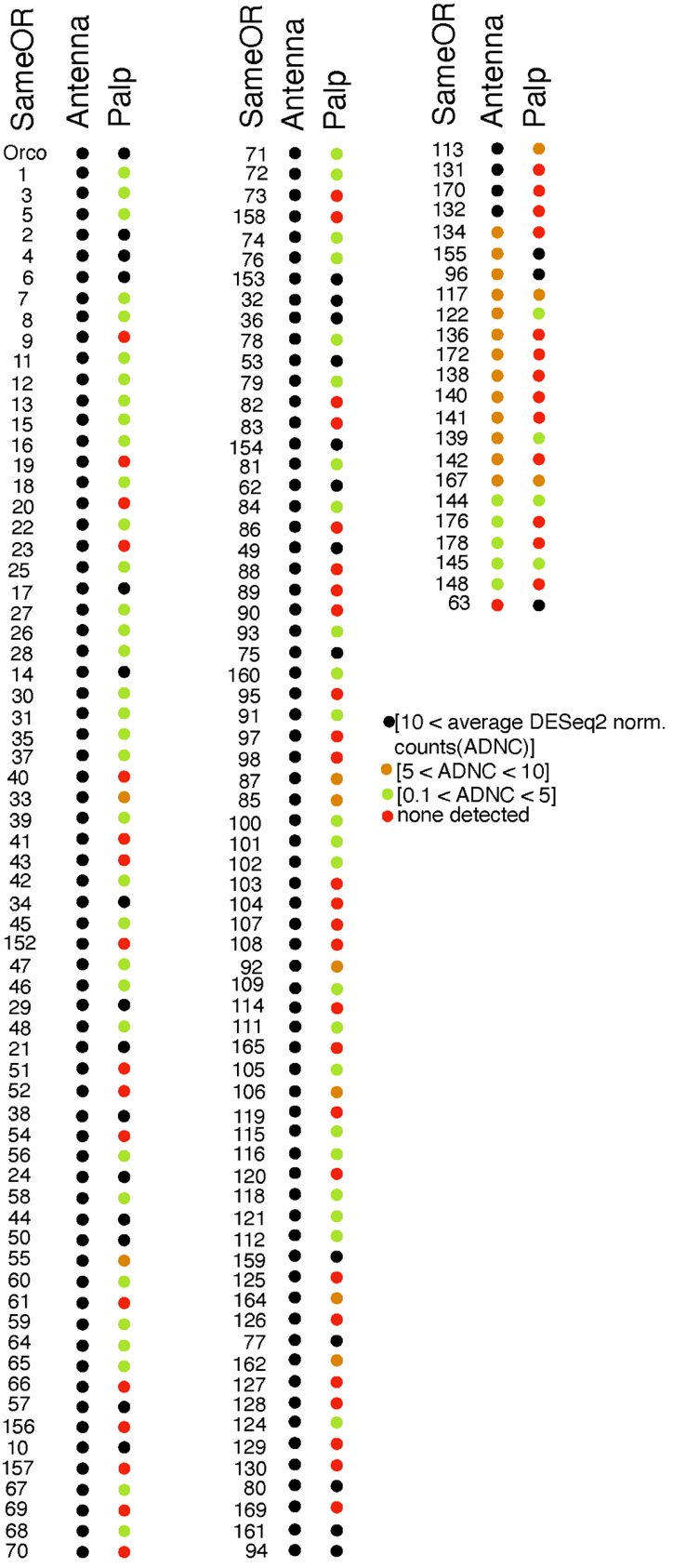
**

**Supplementary Figure 3.** Enlargement of SameOR’s labels presented in Figure 2a. Colored circles next to each SameOR label indicate expression level: black: 10 < average < ~ 4,300; orange: 5 < average < 10; green: 0 < average < 5 normalized counts. Red: none detected. Light gray lines shown in Figure 2a correspond to the columns in this figure.

**
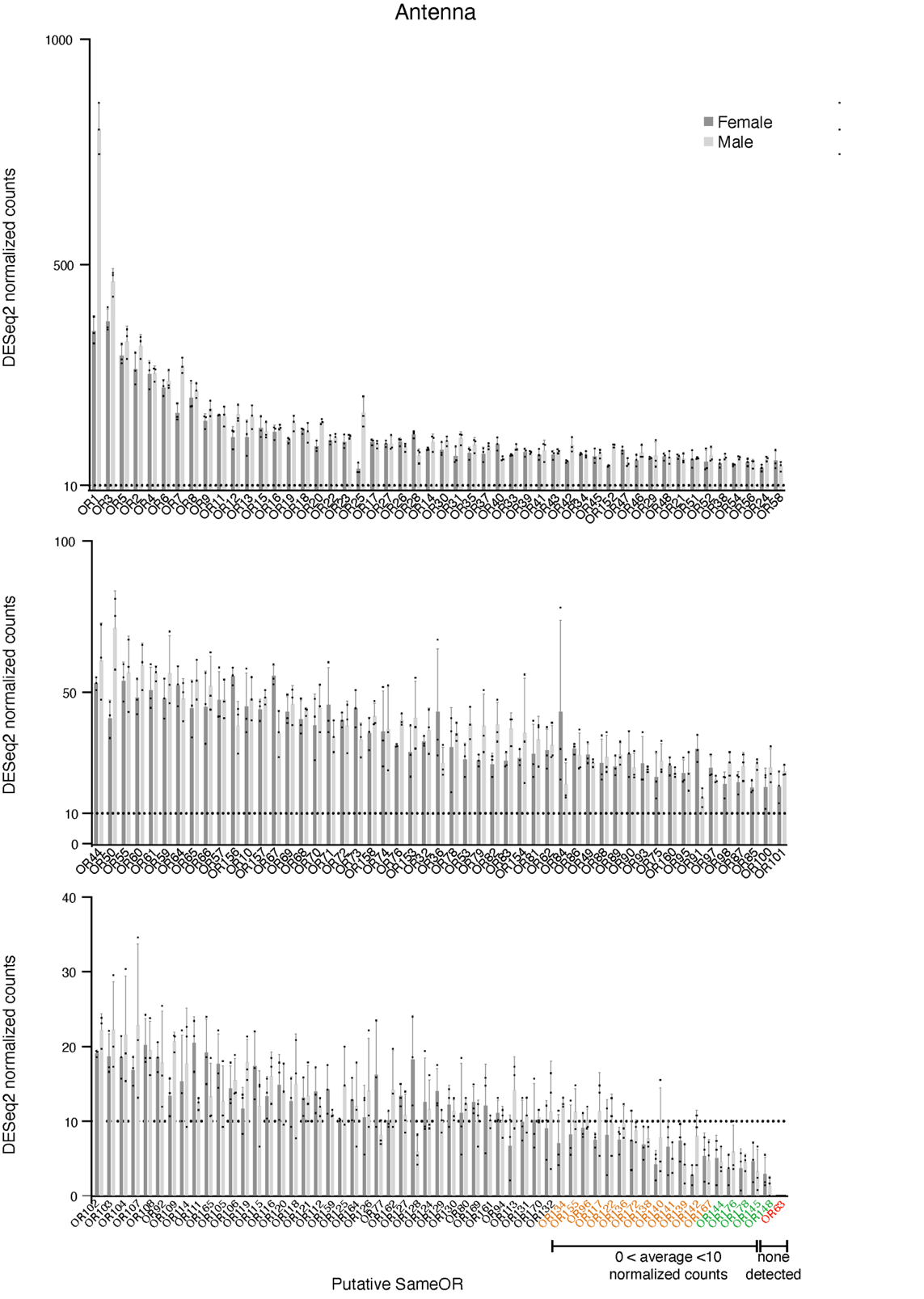
**

**Supplementary Figure 4.** Expression of putative SameORs in antennae of female (dark gray) and male (light gray) locust. RNAseq reads were aligned against the identified putative SameORs and quantified. Quantified values were normalized using DESeq2. Data are organized from highest to lowest normalized counts. Colored labels indicate levels of expression: black: 10 < average < ~ 4,300; orange: 5 < average < 10; green: 0 < average < 5 normalized counts. Red: none detected. Quantification was performed in three replicates per condition. Error bars: standard deviation.

**
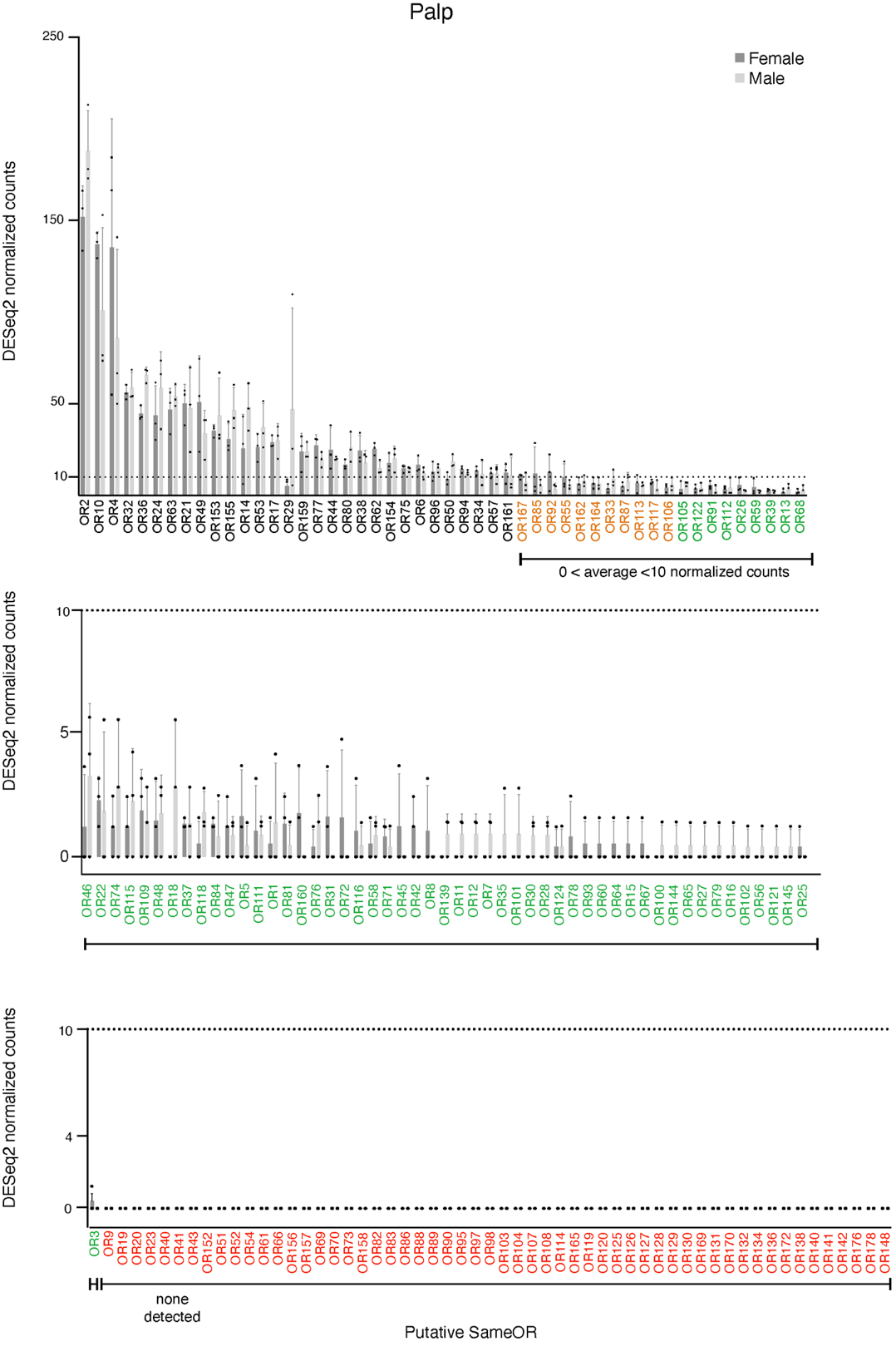
**

**Supplementary Figure 5.** Expression of putative SameORs in palps of female (dark gray) and male (light gray) locust. RNAseq reads were aligned against the identified putative SameORs and quantified. Quantified values were normalized using DESeq2. Data are organized from highest to lowest normalized counts. Colored labels indicate levels of expression: black: 10 < average < ~ 4,300; orange: 5 < average < 10; green: 0 < average < 5 normalized counts. Red: none detected. Quantification was performed in three replicates per condition. Error bars: standard deviation.

**
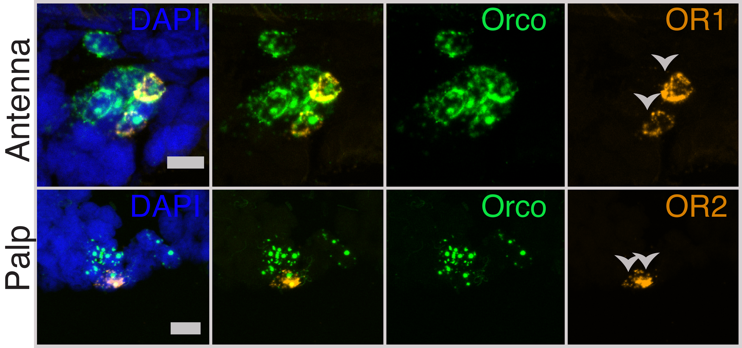
**

**Supplementary Figure 6.** RNAscope *in situ* hybridization of SameOrco with SameOR1 or SameOR2 in antenna or palp sections, respectively. Expression of these ORs was observed only in basiconic sensilla. Two Orco^+^ cells expressing SameOR1 or SameOR2 within the same sensillum are indicated by arrows. Scale bars: 10 μm.

**
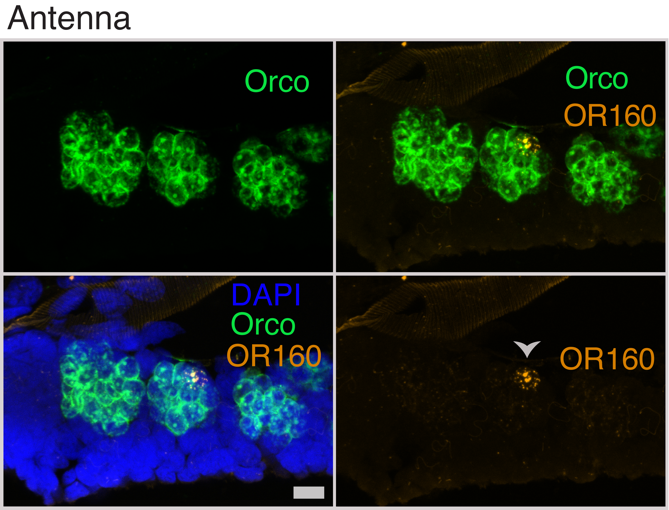
**

**Supplementary Figure 7.** RNAscope *in situ* hybridization of SameOrco with SameOR160 in antenna sections showing their expression in basiconic sensilla. Scale bars: 10 μm.


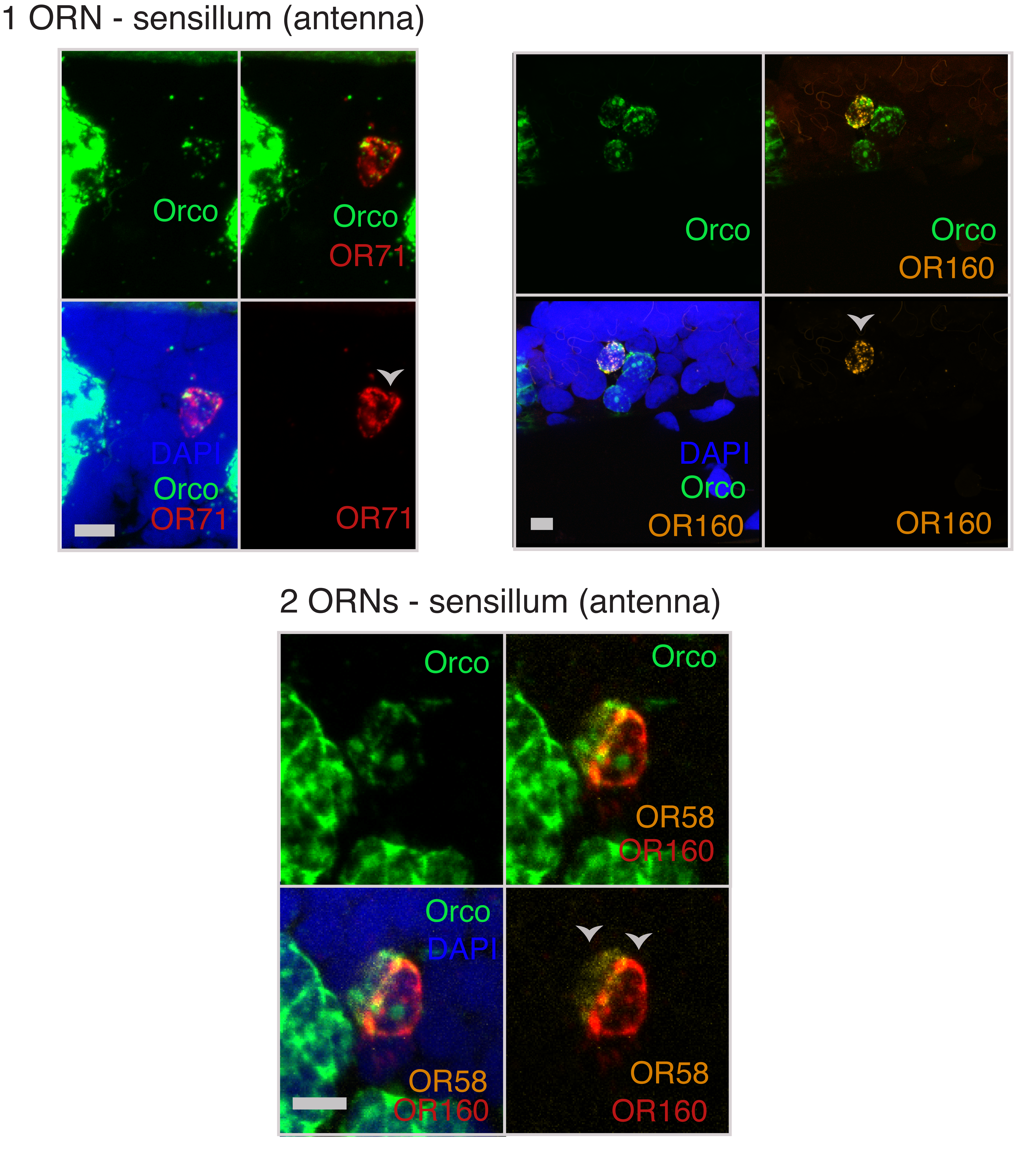


**Supplementary Figure 8.** RNAscope *in situ* hybridization of SameOrco + SameOR71, SameOrco + SameOR160, and SameOrco + SameOR58 + SameOR160 in antenna sections showing their expression in trichoid sensilla. Scale bars: 10 μm.

| **ID Sam transcript targeted** | **Forward** | **Reverse** |
| --- | --- | --- |
| SameGAPDH | CTGCACTACAAATTGCCTGGCACC | TGCAGCTCCAGTTGATGCTGGAAT |
| SameOrco | GTTCGACGCCTCTTCTGGTATGGG | GCGGCTTCATGATCTCCTTGAGGT |
| SameOR1 | AGTGTTCCTATATTGCTGGGCCGC | AGGCTTCCCTGTCAATGGGGTAGA |
| SameOR2 | TATACTGTTGGTGCGCTCACGACG | GTGTATATACGGCCTGCGGAGACG |
| SameOR4 | ATGCCTATTCGCTGTGGACCATCG | GCAGCACGAAGATGGTACCCTTGA |
| SameOR5 | GTCACGTCCCAAGTCTTGTCGCTT | CGAGATGCTGGAGTAACAGCACGG |
| SameOR7 | GGATTGGCGGTGATGCTACAGGAA | CTGTGACGCCTGGAGCATGAAGAT |
| SameOR9 | TAGCGTACCTCCAGGACACCATGA | CTTGGCCTCCCGGAATTGGAAGAA |
| SameOR25 | GCAGTATTCCTACTTCGCCGCCAT | TCAGAGAGTTCCCTCCACGCTGAA |
| SameOR28 | ATAGCAGCACGGGAGGCAGATTTC | GATGGCTATGAAGACGCCCGACAA |
| SameOR40 | GGTATTGACTGTCTCCTGGCGTCG | CGACGTCATAGCGACGGGATTCAT |
| SameOR58 | CACCATGTTCAGCACCAACAGCAC | GATGAGCATGCCGGCGAAGTAGAT |
| SameOR60 | GTCGTGGTGATTCTGCTGCTGTTC | TCGATGAACTGGTCGATAGCGGGT |
| SameOR63 | TGGACGCAGGGAGTCTCACCAAAT | AGTGCAGTAGGAAGCAGCTCGGAT |
|  | GCGTTTTTGAGGAATACGGCGGCA | AAGCAGCTCGGATCTGTAGTGGGT |
| SameOR71 | GGATTGGCGGTGATGCTACAGGAA | CTGTGACGCCTGGAGCATGAAGAT |
|  | CAGGATCTTTCTGTTGACGGGGC | CAGGATGACCCGTTTCCCGACTTT |
| SameOR160 | GTGGGATCGCTCTGTACTGCATC | TAAGACCCATTCAGCACCTGCAC |

**Supplementary Table 3.** Gene-specific primers used for RT-PCR and Sanger sequencing.

**Supplementary Fasta 1.** CDS used for annotation of SameORs containing known OR-CDS from closely related species (*S. gregaria, L. migratoria, Ceracris nigricornis*, and *Oedaleus infernalis*)

**Supplementary Fasta 2.** Sequences used for annotation containing sequences labeled as OR from NCBI nucleotide database

**Supplementary Fasta 3.** CDS of putative SameORs from Database A and Database B

**Supplementary Fasta 4.** Predicted amino acid sequences from putative SameORs from Database A and Database B

**Supplementary Data 1.** Excel file containing the results of the analyses performed on transcripts included in Database A.

**Supplementary Data 2.** Excel file containing the results of the analyses performed on transcripts included in Database B

**Supplementary Data 3.** Alignments performed in CLUSTALO of predicted protein sequences of Odorant Receptor CO-receptor (ORCO) sequences of five insect species (*D. melanogaster, A. gambiae, M. sexta, S. gregaria, L. migratoria*) and the deduced amino acid sequence of the *S. americana* ORCO identified in this work

**Supplementary Data 4.** Alignments performed in CLUSTALO of all the predicted protein sequences of identified putative SameORs contained in Database A against the previously identified *S. gregaria* and *L. migratoria* OR amino acid sequences
